# Comparison of Health Care Utilization in Different Usual Sources of Care Among Older People With Cardiovascular Disease in China: Evidence From the Study on Global Ageing and Adult Health

**DOI:** 10.3389/ijph.2023.1606103

**Published:** 2024-01-03

**Authors:** Tiange Xu, Ekaterina Loban, Xiaolin Wei, Zhongliang Zhou, Wenhua Wang

**Affiliations:** ^1^ School of Public Policy and Administration, Xi’an Jiaotong University, Xi’an, China; ^2^ Department of Family Medicine, McGill University, Montreal, QC, Canada; ^3^ Dalla Lana School of Public Health, University of Toronto, Toronto, ON, Canada

**Keywords:** usual source of care, cardiovascular disease, health care utilization, primary care, China

## Abstract

**Objectives:** To compare the health care utilization in different usual sources of care (USCs) among the elderly population with cardiovascular disease in China.

**Methods:** Cross-sectional data for 3,340 participants aged ≥50 years with cardiovascular disease from Global AGEing and Adult Health (2010)-China were used. Using the inverse probability of treatment weighting on the propensity score with survey weighting, combined with negative binomial regression and logistic regression models, the correlation between USCs and health care utilization was assessed.

**Results:** Patients using primary care facilities as their USC had fewer hospital admissions (IRR = 0.507, 95% CI = 0.413, 0.623) but more unmet health needs (OR = 1.657, 95% CI = 1.108, 2.478) than those using public hospitals. Patients using public clinics as their USC had higher outpatient visits (IRR = 2.188, 95% CI = 1.630, 2.939) than the private clinics’ group.

**Conclusion:** The difference in inpatient care utilization and unmet health care needs between public hospitals and primary care facilities, and the difference in outpatient care utilization between public and private clinics were significant. Using primary care facilities as USCs, particularly public ones, appeared to increase care accessibility, but it still should be strengthened to better address patients’ health care needs.

## Introduction

Population ageing is considered to be challenging for most countries in the world, particularly for China – the country with the world’s largest aging population [1, 2]. In China, the elderly population is estimated to rise to 400 million, accounting for 26.9% of the total population by 2050 [3]. With the accelerated population aging, the incidence and burden of non-communicable diseases (NCDs) have continued to increase in China. The prevalence rate of NCD increased from 157.4‰ to 342.9‰ from 2008 to 2018 [4]. According to the World Health Organization (WHO), about 68.6% of the disease burden is attributable to NCD, China’s rapid population aging is expected to increase the NCD burden by at least 40% by 2030 if the NCD is not controlled effectively [5].

Cardiovascular disease (CVD) as the main NCD constitutes the leading cause of death globally, representing 32% of all global deaths as well as being the cause of more than 40% of deaths in the Chinese population [6–8]. Over the past decades, the prevalence and mortality of CVD have increased dramatically in China. According to recent research, the prevalence cases of CVD increased from 40.57 million in 1990 to 93.81 million in 2016, and the age-standardized prevalence of CVD rose from 5,266 to 6,037 per 100,000 in the same time period. Meanwhile, the mortality rate increased from 174 to 309.33 per 100,000 in rural areas and from 213 to 265.11 per 100,000 in urban areas [9]. As the major NCD in China, CVD has posed unprecedented challenges to Sustainable Development Goals (SDG target 3.4) as well as NCD prevention and control in China.

Usual source of care (USC) is conceptualized as a regular place (doctor’s office, clinic, health center, or other places) or medical professional that a person visits most often for health care when needed [10]. A growing body of literature suggests that having a USC is associated with improved access to health care, decreased medical expenditures, appropriate preventive and treatment services for chronic conditions, and lower rates of unmet health needs [11–14]. Within the current health care delivery system, Chinese patients have several choices of USC: public hospitals and primary care facilities, which are the main health care providers [15]. Most public hospitals are owned by the government, with revenues derived from government subsidy (8.97% of their total revenues) and health care fees [16]. They are perceived to have higher capacity (including adequate numbers of health care professionals, high level medical equipment and technology) at a higher price and provide both specialist and primary care [17]. Primary care facilities, including township health centers, community health centers, public clinics, and private clinics, are regarded as the health care system gatekeepers and are responsible for delivering primary care and public health services. They are predominantly subsidized by the government, with the exception of private clinics, and provide care at a lower cost, but have limited health care capacity [17].

The role of community-based primary care in the prevention and control of CVD has been emphasized in many countries. For example, the American Heart Association Guide for Improving Cardiovascular Health at the Community Level recommends implementing prevention and treatment at the community level [18]. The 2016 European Guidelines on CVD Prevention in Clinical Practice highlight the role of primary care in CVD prevention and management and suggest that the general practitioner should be at the core of long-term health care provision [19]. To reduce health disparities between different populations, the Korean government has proposed to implement a community-based health care services program for chronic disease patients [20]. From 2009 to date, the Chinese government has enacted a series of health policies to build primary care capacity, such as family-doctor-contract services system, to provide basic clinical care, public health services, and health management services for the residency, especially for chronic disease patients.

Primary care facilities are supposed to be the optimal USC for NCD patients in China. However, a recent study found that Chinese patients with chronic disease preferred hospitals as their health care providers [21, 22]. Studies have also revealed that health care utilization was different among different medical institutions. For example, one study of stroke patients indicated that the annual number of stroke-related outpatient visits (primary: 0.42, secondary: 0.59, tertiary: 0.41) and hospital admissions (primary: 0.13, secondary: 0.33, tertiary: 0.34) per patient in primary care facilities were fewer than that in hospitals [23]. In addition, variations in costs tended to be driven by the level of medical institutions [22]. Patients with stroke in hospitals incurred higher costs per outpatient visit (primary: 339 yuan, secondary: 421 yuan, tertiary: 533 yuan) and hospital admission (primary: 6,262 yuan, secondary: 9,250 yuan, tertiary: 18,374 yuan) than that in primary care facilities [23].

Although the benefit of the USC has been widely recognized internationally, few studies have been conducted in China, especially on CVD. Based on the data collected by the WHO from eight provinces in China, our study aimed to compare health care utilization in three types of USCs among the elderly with CVD. Our study complements existing research on the USC and fills the gap in knowledge regarding the difference in health care utilization in different USCs among the elderly living with CVD. This study has important implications for broader chronic disease management in China.

## Methods

### Data Source

The WHO Study on global AGEing and adult health (SAGE) is a longitudinal study with nationally representative samples of people aged ≥50 years old and comparison samples of people aged 18–49 in six low- and middle-income countries, including China [24–27]. Based on the multi-stage cluster sampling design, face-to-face interviews combined with standardized questionnaires were conducted to collect information on socio-demographic characteristics, health risk factors, chronic conditions, health service utilization, and patient responsiveness. The sampling procedure in China consisted of four steps. Step 1: 31 provinces were divided into eastern, central, and western areas in terms of geographic area and socioeconomic level. Step 2: Shanghai, Zhejiang, Guangdong, and Shandong from the eastern area, Hubei and Jilin from the central area, Yunnan and Shaanxi from the western area were randomly selected. Step 3: one county in the rural area and one district in the urban area from national Death Surveillance Points in each province were selected. Step 4: Four townships or communities per country/district, two villages or enumeration areas per township/community, two residential blocks per village/enumeration area, and 42 households per residential block were selected. A total of eight provinces, 16 strata, 64 townships/communities, 128 villages/enumeration areas, 256 residential blocks, and 10,752 households were sampled. All persons in these households were invited to participate in the survey. Finally, 14,811 participants (1,636 individuals aged 18–49 and 13,175 individuals aged 50 years and above) were included, with an overall response rate of 93%.

### Study Population

This is a secondary analysis using the WHO SAGE-China data. We selected our study population in the following steps. First, 4,150 participants aged 50 years old and over with CVD (stroke, angina, and hypertension) were selected. Second, only participants who reported their USC as public hospitals or primary care facilities were selected. Over 92.27% of respondents in the WHO SAGE-China survey reported their USC as either public hospitals or primary care facilities. Respondents with missing values in key covariates (gender, age, and education) were included since covariates had nonzero measurement values in at least 80% [28]. In total, 3,340 eligible participants who were 50 years old and above, had CVD, and reported their USC as either public hospitals or primary care facilities were included in our final analysis. Of note, the participants in the WHO SAGE-China study were selected using a randomized sampling method, and inverse probability of treatment weighting (IPTW) on the propensity with survey weighting was adopted to estimate the difference in health care utilization among different types of USCs. Therefore, our results can provide useful information for the whole patient population 50 years old and above living with CVD in China.

### Measurements

#### Outcome Measure

Three indicators were used to measure health care utilization: 1) the number of outpatient visits, which was measured using the question: “In total, how many times did you receive health care or consultation in the last 12 months?”; 2) the number of hospital admissions, which was measured with the question: “Over the last 12 months, how many times were you a patient in a hospital/long-term care facility for at least one night?”; and 3) unmet health needs, based on the question: “The last time you needed health care, did you get health care?”, was recorded as a “yes” or “no.”

#### Usual Source of Care

From the WHO SAGE Survey, the USC was measured by one item: “Thinking about health care you needed in the last 3 years, where did you go most often when you felt sick or needed to consult someone about your health?” As mentioned above, only participants who reported public hospitals or primary care facilities as their USC were selected. Primary care facilities included both public and private clinics by ownership.

#### Control Variables

We selected control variables for our regression models mainly based on Andersen’s Behavioral Model while considering previous relevant studies [29]. In our analysis, factors that influenced health care utilization were grouped into three categories. 1) Predisposing factors: gender, age, marital status, and education. Age was divided into four groups: 50–59 years old, 60–69 years old, 70–79 years old, and 80-plus years old. Marital status was dichotomized into single versus current partnership. Education was split into four categories: illiterate, primary school, secondary school, and high school or above. 2) Enabling factors: residency, insurance status, and income quintile. Residency included urban and rural. Insurance status was a binary variable: yes or no. Income quintile was broken up into five categories: quintile 1 represented the poorest household category and quintile 5 represented the richest household category, which was based on a possession of a set of household assets and a number of dwelling characteristics. 3) Need factors: body mass index (BMI), activities of daily living (ADLs), instrumental activities of daily living (IADLs), depression, and multimorbidity. BMI was defined as four grades: underweight, normal weight, overweight, and obesity according to the body mass index using the WHO criteria [30]. ADLs consisting of 16 items were classified as a dichotomous variable according to whether participants reported limitation in one and above ADLs (yes) and 0 (no) otherwise. IADLs using 5 items were grouped into a binary variable (no or yes): no deficiency (less than or equal to 3 limitations) and having deficiency (more than 3 limitations). Depression (yes or no), derived from 18 items, was used to measure one’s mental health. People were asked if they had been diagnosed with any of the following chronic diseases: angina, arthritis, stroke, diabetes, chronic lung disease, asthma, depression, and hypertension. Based on the number of chronic diseases, multimorbidity variable was divided into two categories: no (one) and yes (two and above) [31].

#### Statistical Analysis

The data analysis was conducted in March 2023. Descriptive statistics were used to report participant characteristics. Chi-square tests were applied to examine the differences in participant characteristics with different USC.

Then, outcome indicators, including the number of outpatient visits and hospital admissions, and unmet health needs, were used to divide the whole population into three samples. For example, 3,313 patients who responded to question 1 of the outcome measure (in total, how many times did you receive health care or consultation in the last 12 months), were regarded as the outpatient sample set. Thus, six different regression models were created, two for each sample.

Next, IPTW on the propensity score in combination with survey weighting was performed to control for differences in individuals’ characteristics when comparing health care utilization among different types of USCs. IPTW combing survey weighting not only can yield unbiased effect estimates, but also can generalize the estimated effect to older people with CVD in China [32–34]. Firstly, logistic regression model using the survey weight as one of the covariates was built to estimate the propensity scores. Secondly, the balance of propensity scores across different USCs and the balance of covariates across different USCs within blocks of propensity scores were assessed. As a result, the mean propensity scores were not different for USC, and the balancing property for covariates was balanced in all blocks. Thirdly, the IPTW combing survey weighting was built to conduct the propensity score weight. After weighting, the different USCs in six models were balanced in the weighted samples (standardized differences in the weighted samples <10%).

Afterward, considering the over-dispersed distribution of the number of outpatient visits and hospital admissions, multivariable negative binomial regression models with the calculation of robust standard errors were built to compare the outpatient and inpatient health care utilization among different USCs. As the unmet health needs were a binary variable (yes/no), a multivariable logistic regression model was constructed.

Finally, to explore the differential effect in population groups, we did subgroup analyses stratified by residency, using the same regression analyses. The results were consistent with the findings of our main analysis. Statistical significance was set at *p* < 0.05. The data analysis was conducted using STATA version 15.1.

## Results

### Participant Characteristics

Of 3,340 CVD patients in our study, 2,197 (65.78%) identified public hospitals as their USC, whereas 619 (18.53%) identified public clinics as their USC, and 524 (15.69%)—private clinics. Table 1 provides a detailed description of the socio-demographic characteristics of these patients. Female participants were less likely to choose public hospitals and public clinics as their USC (55.53% and 58.16%, respectively, compared with 62.40% choosing private clinics). Older participants tended to choose public hospitals and public clinics as their USC (72.46% and 70.44%, respectively, vs. 63.55% choosing private clinics). Participants with high school or above education tended to use public hospitals (27.54%) as their USC, compared with those using public clinics (13.73%) and private clinics (5.15%). Urban residents preferred to visit public hospitals (75.33%) compared with public clinics (41.52%) and private clinics (31.87%). Most participants who used public hospitals (86.95%) and public clinics (93.84%) as their USC had higher health insurance coverage rates than those reporting private clinics (79.96%) as their USC. Nearly one-third of participants (28.07%) who used public hospitals as their USC were in the richest income quintile, while more participants using private clinics (34.23%) and public clinics (19.74%) were in the poorest income quintile. Most participants who usually visited private clinics for health care had ADLs (81.11%) and IADLs (13.36%) compared with those visiting public hospitals (ADLs:70.23%; IADLs:9.15%) and public clinics (ADLs:71.41%; IADLs:13.25%). The multimorbidity rate was the highest in public hospitals (60.90%) than in private clinics (50.57%) and public clinics (52.34%). There were no obvious differences by marital status, BMI, and depression among participants with different USC. The baseline characteristics in outpatient, inpatient, and unmet health needs samples among different types of USCs are presented in the Appendices (Supplementary Tables S1–S5).

**TABLE 1:** Distribution of participant characteristics by different types of USCs. (China, 2010).

Characteristics	Total (*n* = 3,340)	Public hospitals (*n* = 2,197)	Private clinics (*n* = 524)	Public clinics (*n* = 619)	*p*-value^a^
Gender					
Male	1,433 (42.90)	977 (44.47)	197 (37.60)	259 (41.84)	0.014
Female	1907 (57.10)	1,220 (55.53)	327 (62.40)	360 (58.16)	
Age					
50–59 years old	979 (29.31)	605 (27.54)	191 (36.45)	183 (29.56)	<0.001
60–69 years old	1,097 (32.84)	694 (31.59)	185 (35.31)	218 (35.22)	
70–79 years old	1,006 (30.12)	710 (32.32)	116 (22.14)	180 (29.08)	
≥80 years old	257 (7.72)	188 (8.56)	32 (6.11)	38 (6.14)	
Marital status					
Single	631 (18.90)	404 (18.40)	118 (22.52)	109 (17.64)	0.065
Current partnership	2,707 (81.10)	1,792 (81.60)	406 (77.48)	509 (82.36)	
Education					
Illiterate	826 (24.73)	438 (19.94)	208 (39.69)	180 (29.08)	<0.001
Primary school	1,136 (34.01)	671 (30.54)	214 (40.84)	251 (40.55)	
Secondary school	663 (19.85)	485 (22.8)	75 (14.31)	103 (16.64)	
High school or above	715 (21.41)	603 (27.45)	27 (5.15)	85 (13.73)	
Residency					
Urban	2,079 (62.25)	1,655 (75.33)	167 (31.87)	257 (41.52)	<0.001
Rural	1,261 (37.75)	542 (24.67)	357 (68.13)	362 (58.48)	
Insurance					
No	427 (12.87)	284 (13.05)	105 (20.04)	38 (6.16)	<0.001
Yes	2,891 (87.13)	1,893 (87.95)	419 (79.96)	579 (93.84)	
Income quintile					
Poorest	560 (16.86)	259 (11.88)	179 (34.23)	122 (19.74)	<0.001
Q2	565 (17.01)	290 (13.30)	156 (29.83)	119 (19.26)	
Q3	674 (20.30)	457 (20.96)	85 (16.25)	132 (21.36)	
Q4	772 (23.25)	562 (25.78)	76 (14.53)	134 (21.68)	
Richest	750 (22.58)	612 (28.07)	27 (5.16)	111 (17.96)	
BMI					
Underweight	77 (2.31)	47 (2.14)	15 (2.86)	15 (2.42)	0.307
Normal weight	1,666 (49.88)	1,099 (50.02)	277 (52.86)	290 (46.85)	
Overweight	1,164 (34.85)	762 (34.68)	177 (33.78)	225 (36.35)	
Obesity	433 (12.96)	289 (13.15)	55 (10.50)	89 (14.38)	
ADLs					
No	930 (27.84)	654 (29.77)	99 (18.89)	177 (28.59)	<0.001
Yes	2,410 (72.16)	1,543 (70.23)	425 (81.11)	442 (71.41)	
IADLs					
No	2,987 (89.43)	1,996 (90.85)	454 (86.64)	537 (86.75)	0.001
Yes	353 (10.57)	201 (9.15)	70 (13.36)	82 (13.25)	
Depression					
No	3,266 (97.78)	2,156 (98.13)	506 (96.56)	604 (97.58)	0.084
Yes	74 (2.22)	41 (1.87)	18 (3.44)	15 (2.42)	
Multimorbidity					
No	1,413 (42.31)	859 (39.10)	259 (49.43)	295 (47.66)	<0.001
Yes	1927 (57.69)	1,338 (60.90)	265 (50.57)	324 (52.34)	

Distribution reported excludes those with missing data.

^a^ *p*-value based on Chi-square test.

USC, usual source of care; BMI, body mass index; ADLs, activities of daily living; IADLs, instrumental activities of daily living limitation; n, number.

### Health Care Utilization by Type of USC

Information on health care utilization by different types of USCs is displayed in Table 2. Patients using public hospitals as their USC had more outpatient visits and hospital admissions, but a lower likelihood of unmet health needs (mean [SD] outpatient visits, 6.25 [11.86]; mean [SD] hospital admissions, 0.31 [0.66]; number [proportion] unmet health needs, 92 [4.20]) than those using primary care facilities (mean [SD] outpatient visits, 3.58 [6.46]; mean [SD] hospital admissions, 0.18 [0.52]; number [proportion] unmet health needs, 124 [10.88]). Further analysis of primary care facilities indicated that patients identifying public clinics as their USC tended to report more outpatient visits and a higher likelihood of unmet health needs (mean [SD] outpatient visits, 4.49 [7.46]; number [proportion] unmet health needs, 39 [6.32]) than those using private clinics (mean [SD] outpatient visits, 2.50 [4.81], number [proportion] unmet health needs, 84 [16.15]).

**TABLE 2:** Health care utilization by different types of USCs. (China, 2010).

Health care utilization	Total	USC		*p*-value	Total	USC		*p*-value
		Public hospitals	Primary care facilities			Private clinics	Public clinics	
Number of outpatient visits^a^ (mean, SD)	5.33 (10.40)	6.25 (11.86)	3.58 (6.46)	<0.001	3.56 (6.46)	2.50 (4.81)	4.49 (7.46)	<0.001
Number of hospital admissions^b^ (mean, SD)	0.27 (0.62)	0.31 (0.66)	0.18 (0.52)	<0.001	0.18 (0.52)	0.20 (0.49)	0.17 (0.54)	0.177
Unmet health needs^b^ (n, %)	216 (6.48)	92 (4.20)	124 (10.88)	<0.001	123 (10.82)	84 (16.15)	39 (6.32)	<0.001

^a^ Rank sum test was used.

^b^ Chi-square test was used.

USC, usual source of care; SD, standard deviation; n, number.

**FIGURE 1:**
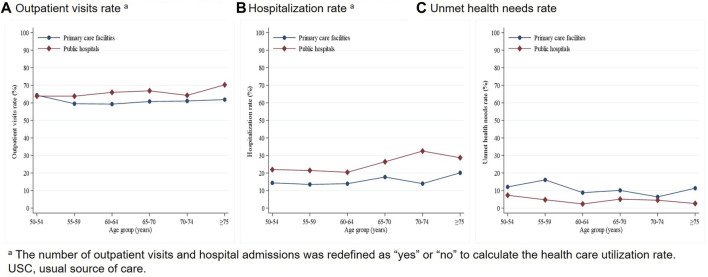
Trend of health care utilization by age and USC (public hospitals and primary care facilities). (China, 2010).

**FIGURE 2:**
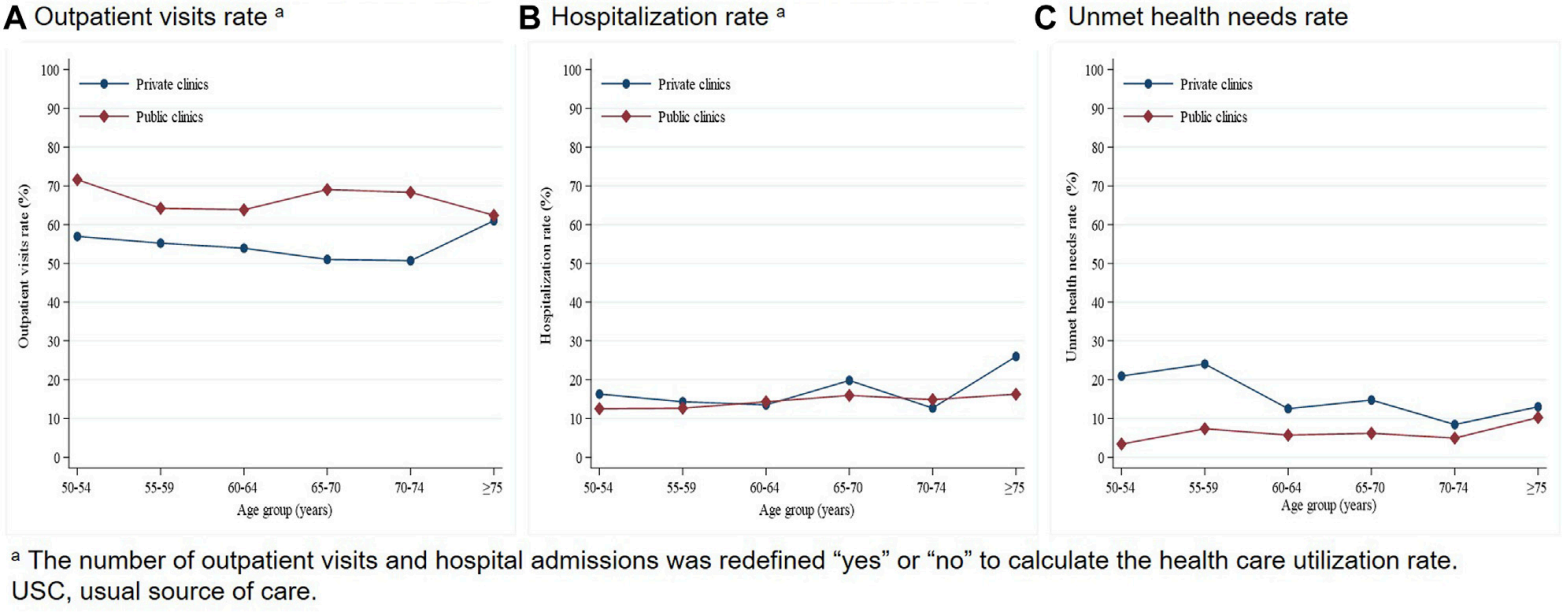
Trend of health care utilization by age and USC (private clinics and public clinics) (China, 2010).

We conducted further analysis to determine the distribution of the rate of outpatient visits, hospitalization, and unmet health needs for different types of USCs by age, given that age is an important determinant of CVD, USC, and health care utilization (Figures 1A–C, 2A–C). The number of outpatient visits and hospital admissions was redefined as “yes” or “no” to calculate the health care utilization rate. In general, the outpatient visits, hospitalization, and unmet health needs rates for participants aged 50 years old and over with CVD were 64.43%, 22.33%, and 6.48% respectively. Older patients using public hospitals as their USC had higher outpatient visits and hospitalization rates, and lower unmet health needs rates compared with those using primary care facilities as their USC. Further analysis at the level of primary care facilities revealed that older patients using public clinics as their USC tended to report higher outpatient visits and lower unmet health needs rates compared with those using private clinics as their USC. However, there were no obvious differences in hospitalization rates between public clinics and private clinics in this age group.

### USC and Health Care Utilization

The standardized differences in both unweighted and weighted samples were shown in Supplementary Tables S6, S7 in the Appendices. After IPTW combing with sampling weight, the standardized differences of covariates between public hospitals and primary care facilities, private clinics and public clinics were less than 10%, which denoted that the baseline characteristics between different USCs were balanced.

Table 3 displays the results of multivariable regression using weighted data. The differences between public hospitals and primary care facilities in terms of hospital admissions and unmet health needs were statistically significant. The probability of unmet health needs for patients using primary care facilities as their USC was higher (OR = 1.657, 95% CI = 1.108, 2.478), but the number of hospitals admissions (IRR = 0.507, 95% CI = 0.413, 0.623) was lower compared with patients using public hospitals as their USC. Table 3 also contains the results of multivariable regression between USC (private clinics and public clinics) and health care utilization using weighted data. Compared with individuals who used private clinics as their USC, individuals who used public clinics as their USC had more outpatient visits (IRR = 2.188, 95% CI = 1.630, 2.939). There were no statistically significant differences in hospital admissions and unmet health needs. Additionally, our study distinguished patients residing in urban and rural areas and compare the health care utilization in three types of USCs among two groups of patients (Supplementary Table S8 in the Appendices). The correlation between USCs and health care utilization appeared to be similar across urban and rural areas, except for the correlation between public clinics, private clinics, and inpatient care utilization.

**TABLE 3:** Multivariable regression analysis for the health care utilization by different types of USCs after weighting. (China, 2010).

Characteristics	Outpatient visits model		Hospital admissions model		Unmet health needs model	
	IRR (95% CI*)*	*p*-value	IRR (95% CI)	*p*-value	OR (95% CI)	*p*-value
USC (ref. = public hospital)^a^						
Primary Care Facility	0.955 (0.708, 1.289)	0.761	0.507 (0.413, 0.623)	<0.001	1.657 (1.108, 2.478)	0.015
USC (ref. = Private clinics)^b^						
Public clinics	2.188 (1.630, 2.939)	<0.001	0.817 (0.566, 1.180)	0.274	0.728 (0.416, 1.274)	0.259

^a^ Using public hospitals and primary care facilities as the USC.

^b^ Using private clinics and public clinics as the USC.

USC, usual source of care; IRR, adjusted relative rate; OR, odds ratio.

## Discussion

This study compared the health care utilization in three types of USCs among Chinese CVD patients aged 50 and over. We found that nearly 70% of older participants with CVD chose public hospitals as their USC rather than primary care facilities. When it comes to health care utilization, the elderly CVD patients using primary care facilities as their USC generally had fewer hospital admissions, but a higher probability of unmet health needs compared with those using public hospitals as their USC. At the primary care level, the elderly CVD patients identifying public clinics as their USC had more outpatient visits compared with those using private clinics as their USC. Using primary care facilities as the USC, particularly public primary care facilities, appeared to increase access to health care to a certain extent.

Our data indicated that nearly 70% of older CVD patients choose public hospitals as their USC rather than primary care facilities, which is consistent with a prior study conducted in China that 69.63% of heart failure patients choose hospitals to receive treatment in preference to primary care facilities [35]. Even China has made remarkable progress in strengthening primary health care, however, the quality of primary health care is still deemed poor, resulting in patients bypassing primary care facilities and seeking health care from hospitals for chronic conditions [36–38]. This preference for hospitals was contrary to international recommendations to prevent and control CVD at the level of primary care facilities [18–20]. The Chinese government still needs to struggle to ensure the quality of primary health care in NCD prevention and control.

Health care was largely underused by CVD patients, particularly outpatient services. In our study, more than one-third of patients had not been treated as outpatients in the past year, and less than a quarter of patients had been hospitalized over the past year. Meanwhile, the mean number of outpatient visits per year was 5.33 times, which was lower than that suggested in the Chinese guidelines for hypertension management, of once every two or four weeks, particularly for patients with poorly controlled blood pressure [39]. Other clinical guidelines also require regular monitoring of blood lipids and medications for patients with CVD in prevention [40, 41]. This underuse might lead to poorly adhering to treatment guidelines, then increase hospitalization and health care cost [42].

In the analysis of public hospitals and primary care facilities as USC, we found that patients using primary care facilities as their USC had fewer hospital admissions than those who adopted public hospitals as their USC, and the difference in outpatient visits was not significant. These results suggested that primary health care fulfilled certain functions in increasing access to health care in older patients with CVD, which is likely to reflect the achievement of China’s primary health care system reform with core responsibilities in preventing and managing chronic disease [38, 43]. This finding can support the utilization of primary care facilities as the USC to prevent and control CVD. However, among elderly people with CVD, those who used primary care facilities as their USC had a comparatively higher probability of unmet health needs than those using public hospitals as their USC. This difference in unmet health needs can be explained by the quality of health care provided in these medical institutions [44, 45]. Public hospitals, in terms of service capacity (including the availability of health professionals and equipment) and diagnoses of severe or complex diseases, have relatively high quality [17, 31]. In comparison, although some progress has been made with increased financial investments in primary care, primary care facilities frequently struggle with structural characteristics, incentives and policies, and other serious challenges, widespread gaps in the quality of primary health care still exist [36, 38, 46]. One study revealed that for patients with dysentery or unstable angina symptoms, village doctors completed only 18% of the suggested questions and correctly diagnosed only 26% of patients’ conditions [47]. Multimorbidity may also be associated with this difference in health care utilization between public hospitals and primary care facilities. Several studies have shown that multimorbidity has a significant influence on the likelihood of accessing health care, particularly for individuals living with CVD [48–50]. In our study, no matter whether patients choose public hospitals or primary care facilities as their USC, more than half of them suffered from multimorbidity. Due to the difference in quality of care, public hospitals could better meet their health care needs than primary care facilities.

At the primary health care level, we found that those who chose public clinics as their USC had more outpatient visits than those who used private clinics as their USC. This gap seems to be largely mediated by the quality of primary health care. A national study conducted in China, aiming to rate the care quality of public and private clinics based on patient-perceived quality, revealed that public clinics were rated more favorably in prompt attention, communication, autonomy, dignity, and confidentiality [31]. In the current health system reform, in addition to increasing substantial financial investment and infrastructure building in primary health care, the Chinese government also encourages partnerships between hospitals and primary care facilities, especially public primary care facilities, such as establishing integrated health care delivery system based on primary health care, to strengthen local primary health care capacity and hence prevent and manage chronic diseases [36, 38]. Previous research also demonstrated that private primary care facilities tended to be poorly regulated and suffered from low quality of prescribing, which was related to limited government subsidies, limited availability of essential medicines, and their for-profit orientation [31, 51, 52]. Moreover, higher public trust and stronger preference for public primary health care facilities might be considered a factor influencing health care utilization [31, 51]. A previous study in three Chinese provinces showed that only 8% of individuals stated that physicians in private clinics had better skills than that in public clinics, and just 29% of individuals reported they would prefer to see a private doctor than a public doctor [51]. Thus, compared with private clinics, public clinics have a higher quality of continuity and coordination in CVD management with a higher degree of trust, leading to greater utilization of outpatient services by patients who identified public clinics as USC. Notably, in China, progress has been made toward the prevention and control of NCD in public primary health care, but substantial opportunities for primary health care improvement remain.

Our findings have important policy implications for CVD prevention and control. Firstly, although primary care facilities, as USC, play a certain role in increasing access to health care in older patients with CVD, suboptimal quality persists. It is necessary to call upon the government to pay more attention to the improvement of primary care quality. More health care resources, especially highly qualified health care workers, should be allocated to primary care facilities to narrow the capacity gap between primary care facilities and hospitals [37, 38, 53]. Secondly, primary care facilities are at a disadvantage in the tiered health care delivery system, which rendered them difficult to function as gatekeepers. It is essential to strengthen the coordination between primary care facilities and hospitals to establish an integrated delivery system, such as improving the bidirectional referral mechanism, changing the provider payment mechanism to pay according to the size of the population served and the quality of care delivered, integrating and sharing electronic patients’ records [38]. Thirdly, to shift citizens’ health care demands toward primary health care, the Chinese government should consider organizing large-scale health education activities to improve nationwide understanding of primary health care function and its importance in the prevention and control of NCD, thereby guiding citizens to develop appropriate health care seeking behaviors [36].

This study has several limitations. First, the SAGE-China data did not cover all types of CVDs. Further studies analyzing the relationship between USC and health care utilization among patients with other CVD are needed. Second, our study used cross-sectional data in 2010 because the second round (2014–2015) of SAGE-China data is still in the process of collation and not available. We could not infer causality between USC and health care utilization. However, Although the data is relatively old, residents’ health care utilization pattern has not changed significantly in the past years [15, 36, 46]. The outpatient visits and hospital admissions at primary care facilities (outpatient visits: 61.72% in 2009, 50.2% in 2021; hospital admissions: 31.01% in 2009, 14.5% in 2021) kept decreasing relative to those at hospitals (outpatient visits: 34.97% in 2009, 45.8% in 2021; hospital admissions: 64.3% in 2009, 81.5% in 2021) from 2009 to 2021. Moreover, the multistage cluster sampling design and using IPTW with survey weighting could help us generalize the estimated effect to the elderly with CVD in China. Considering these reasons, we maintain that our analysis still could provide new insight into CVD prevention and control for the whole elderly population in China. Third, China has established a three-tier health care delivery system containing tertiary hospitals, secondary hospitals, and primary care facilities. In our study, USC was divided into two categories: public hospitals and primary care facilities. We could not clearly distinguish whether the public hospital is a secondary hospital or a tertiary hospital. Further research could compare the health care utilization among different health facility level.

### Conclusion

Our results demonstrated that there was a significant difference in inpatient care utilization and unmet health care needs between primary care facilities and public hospitals. Also, the difference in outpatient care utilization between public and private clinics was found. Primary health care facilities, especially public primary care facilities, fulfilled certain functions in increasing access to health care among the elderly with CVD. But primary care facilities still offer certain deficiencies in terms of meeting patients’ health care needs compared with public hospitals. In the context of China, public primary care facilities may be the optimal USC for the public living with chronic disease, but the quality of care needs to be further strengthened, which is consistent with China’s health care system reform priorities.

## Abbreviations

CVD, Cardiovascular Disease; USC, Usual Source of Care; WHO, World Health Organization; SAGE, Study on Global AGEing and Adult Health; BMI, Body Mass Index; ADL, Activities of Daily Living; IADL, Instrumental Activities of Daily Living; IPTW, Inverse Probability of Treatment Weighting.
