# Supplementary material for: Comparison of Health Care Utilization in Different Usual Sources of Care Among Older People With Cardiovascular Disease in China: Evidence From the Study on Global Ageing and Adult Health
Source: Int J Public Health. 2024 Jan 3;68:1606103. doi: 10.3389/ijph.2023.1606103 (PMC10792126; doi:10.3389/ijph.2023.1606103)
Supplement: Supplementary file 1 [file DataSheet1.docx]

**Supplementary Information**

Table S1 Baseline characteristics between public hospitals and primary care facilities

Table S2 Baseline characteristics between private clinics and public clinics

Table S3 The mean value of outpatient visits among different types of USCs

Table S4 The mean value of hospital admissions among different types of USCs

Table S5 The probability of unmet health needs by different types of USCs

Table S6 Standardized differences between different types of USCs (public hospitals and primary care facilities) before and after weighting

Table S7 Standardized differences between different types of USCs (private clinics and public clinics) before and after weighting

Table S8 Subgroup analyses for the health care utilization between different types of USCs and residency after weighting

Table S1 Baseline characteristics between public hospitals and primary care facilities ^a^ (China, 2010).

| **Characteristics** | **Outpatient visits model, N (%)** | | | |  | **Hospital admissions model, N (%)** | | | |  | **Unmet health needs model, N (%)** | | | |
| --- | --- | --- | --- | --- | --- | --- | --- | --- | --- | --- | --- | --- | --- | --- |
|  | **Total**  **(n =3313)** | **Public hospitals**  **(n =2173)** | **Primary**  **care facilities**  **(n =1140)** | ***P* value** |  | **Total**  **(n =3288)** | **Public hospitals**  **(n =2155)** | **Primary**  **care facilities**  **(n =1133)** | ***P* value** |  | **Total**  **(n =3331)** | **Public hospitals**  **(n =2191)** | **Primary**  **care facilities**  **(n =1140)** | ***P* value** |
| Gender |  |  |  |  |  |  |  |  |  |  |  |  |  |  |
| Male | 1422(42.92) | 966(44.45) | 456(40.00) | 0.014 |  | 1409(42.85) | 959(44.50) | 450(39.72) | 0.008 |  | 1430(42.93) | 975(44.50) | 455(39.91) | 0.011 |
| Female | 1891(57.08) | 1207(55.55) | 684(60.00) |  |  | 1879(57.15) | 1196(55.50) | 683(60.28) |  |  | 1901(57.07) | 1216(55.50) | 685(60.09) |  |
| Age |  |  |  |  |  |  |  |  |  |  |  |  |  |  |
| 50–59 years old | 975(29.43) | 602(27.70) | 373(32.72) | <0.001 |  | 962(29.26) | 593(27.52) | 369(32.57) | <0.001 |  | 977(29.33) | 604(27.57) | 373(32.72) | <0.001 |
| 60–69 years old | 100(32.90) | 688(31.66) | 402(35.36) |  |  | 1084(32.97) | 683(31.69) | 401(35.39) |  |  | 1091(32.75) | 690(31.49) | 401(35.18) |  |
| 70–79 years old | 994(30.00) | 698(32.12) | 296(25.96) |  |  | 993(30.20) | 699(32.44) | 294(25.95) |  |  | 1005(30.17) | 709(32.36) | 296(25.96) |  |
| ≥ 80 years old | 254(7.67) | 185(8.51) | 69(6.05) |  |  | 249(7.57) | 180(8.35) | 69(6.09) |  |  | 258(7.75) | 188(8.58) | 70(6.14) |  |
| Marital status |  |  |  |  |  |  |  |  |  |  |  |  |  |  |
| Single | 625(18.88) | 400(18.42) | 225(19.75) | 0.350 |  | 622(18.93) | 396(18.38) | 226(19.96) | 0.272 |  | 629(18.89) | 403(18.40) | 226(19.84) | 0.314 |
| Current partnership | 2686(81.12) | 1772(81.58) | 914(80.25) |  |  | 2664(81.07) | 1758(81.62) | 906(80.04) |  |  | 2700(81.11) | 1787(81.60) | 913(80.16) |  |
| Education |  |  |  |  |  |  |  |  |  |  |  |  |  |  |
| Illiterate | 819(24.72) | 432(19.88) | 387(33.95) | <0.001 |  | 815(24.79) | 430(19.95) | 385(33.98 | <0.001 |  | 823(24.71) | 437(19.95) | 386(33.86) | <0.001 |
| Primary school | 1130(34.11) | 667(30.69) | 463(40.61) |  |  | 1116(33.94) | 655(30.39) | 461(40.69) |  |  | 1132(33.98) | 668(30.49) | 464(40.70) |  |
| Secondary school | 656(19.80) | 478(22.00) | 178(15.61) |  |  | 651(19.80) | 474(22.00) | 177(15.62) |  |  | 662(19.87) | 484(22.09) | 178(15.61) |  |
| High school or above | 708(21.37) | 596(27.43) | 112(9.82) |  |  | 706(21.47) | 596(27.66) | 110(9.71) |  |  | 714(21.44) | 602(27.48) | 112(9.82) |  |
| Residency |  |  |  |  |  |  |  |  |  |  |  |  |  |  |
| Urban | 2058(62.12) | 1636(75.29) | 422(37.02) | <0.001 |  | 2047(62.26) | 1628(75.55) | 419(36.98) | <0.001 |  | 2077(62.35) | 1653(75.45) | 424(37.19) | <0.001 |
| Rural | 1255(37.88) | 537(27.71) | 718(62.98) |  |  | 1241(37.74) | 527(24.45) | 714(63.02) |  |  | 1254(37.65) | 538(24.55) | 716(62.81) |  |
| Insurance |  |  |  |  |  |  |  |  |  |  |  |  |  |  |
| No | 427(12.97) | 284(13.19) | 143(12.57) | 0.612 |  | 418(12.80) | 275(12.88) | 143(12.64) | 0.847 |  | 427(12.90) | 284(13.08) | 143(12.57) | 0.674 |
| Yes | 2864(87.03) | 1869(86.81) | 995(87.43) |  |  | 2848(87.20) | 1860(87.12) | 988(87.36) |  |  | 2882(87.10) | 1887(86.92) | 995(87.43) |  |
| Income quintile |  |  |  |  |  |  |  |  |  |  |  |  |  |  |
| Poorest | 557(16.91) | 257(11.92) | 300(26.36) | <0.001 |  | 553(16.92) | 253(11.83) | 300(26.53) | <0.001 |  | 558(16.85) | 258(11.87) | 300(26.36) | <0.001 |
| Q2 | 562(17.06) | 288(13.36) | 274(24.08) |  |  | 553(16.92) | 281(13.14) | 272(24.05) |  |  | 564(17.03) | 290(13.34) | 274(24.08) |  |
| Q3 | 670(20.34) | 454(21.06) | 216(19.98) |  |  | 661(20.22) | 446(20.86) | 215(19.01) |  |  | 672(20.29) | 456(20.98) | 216(18.98) |  |
| Q4 | 766(23.25) | 556(25.79) | 210(18.45) |  |  | 760(23.25) | 553(25.87) | 207(18.30) |  |  | 769(23.22) | 559(25.71) | 210(18.45) |  |
| Richest | 739(22.43) | 601(27.88) | 138(12.13) |  |  | 742(22.70) | 605(28.30) | 137(12.11) |  |  | 749(22.61) | 611(28.10) | 138(12.13) |  |
| BMI |  |  |  |  |  |  |  |  |  |  |  |  |  |  |
| Underweight | 77(2.32) | 47(2.16) | 30(2.63) | 0.771 |  | 75(2.28) | 45(2.09) | 30(2.65) | 0.690 |  | 77 (2.31) | 47(2.15) | 30(2.63) | 0.762 |
| Normal weight | 1656(49.98) | 1091(50.21) | 565(49.56) |  |  | 1637(49.79) | 1072(49.74) | 565(49.87) |  |  | 1663(49.92) | 1098(50.11) | 565(49.56) |  |
| Overweight | 1151(34.74) | 749(34.47) | 402(35.26) |  |  | 1148(34.91) | 751(34.85) | 397(35.04) |  |  | 1159(34.79) | 757(34.55) | 402(35.26) |  |
| Obesity | 429(12.95) | 286(13.16) | 143(12.41) |  |  | 428(13.02) | 287(13.32) | 141(12.44) |  |  | 432(12.97) | 289(13.19) | 143(12.54) |  |
| ADLs |  |  |  |  |  |  |  |  |  |  |  |  |  |  |
| No | 921(27.80) | 646(29.73) | 275 (24.12) | 0.001 |  | 917(27.89) | 645(29.93) | 272(24.01) | <0.001 |  | 928(27.86) | 652(29.76) | 276(24.21) | 0.001 |
| Yes | 2392(72.20) | 1527(70.27) | 865(75.88) |  |  | 2371(72.11) | 1510(70.07) | 861(75.99) |  |  | 2403(72.14) | 1539(70.24) | 864(75.79) |  |
| IADLs |  |  |  |  |  |  |  |  |  |  |  |  |  |  |
| No | 2963(89.44) | 1975(90.89) | 988(86.67) | <0.001 |  | 2943(89.51) | 1960(90.95) | 983(86.76) | <0.001 |  | 2979(89.43) | 1991(90.87) | 988(86.67) | <0.001 |
| Yes | 350(10.56) | 198(9.11) | 152(13.33) |  |  | 345(10.49) | 195(9.05) | 150(13.24) |  |  | 352(10.57) | 200(9.13) | 152(13.33) |  |
| Depression |  |  |  |  |  |  |  |  |  |  |  |  |  |  |
| No | 3239(97.77) | 2132(98.11) | 1107(97.11) | 0.062 |  | 3214(97.75) | 2114(98.10) | 1100(97.09) | 0.063 |  | 3257(97.78) | 2150(98.13) | 1107(97.11) | 0.057 |
| Yes | 74(2.23) | 41(1.89) | 33(2.89) |  |  | 74(2.25) | 41(1.90) | 33(2.91) |  |  | 74(2.22) | 41(1.87) | 33(2.89) |  |
| Multimorbidity |  |  |  |  |  |  |  |  |  |  |  |  |  |  |
| No | 1406(42.44) | 853(39.25) | 553(48.51) | <0.001 |  | 1395(42.43) | 845(39.21) | 550(48.54) | <0.001 |  | 1411(42.36) | 858(39.16) | 553(48.51) | <0.001 |
| Yes | 1907(57.56) | 1320(60.75) | 587(51.49) |  |  | 1893(57.57) | 1310(60.79) | 583(34.46) |  |  | 1920(57.64) | 1333(60.84) | 587(51.49) |  |

^a^ Distribution reported excluded those with missing data. USC, usual source of care; BMI, body mass index; ADLs, activities of daily living; IADLs, instrumental activities of daily living limitation; N, number.

Table S2 Baseline characteristics between private clinics and public clinics ^a^ (China, 2010).

| **Characteristics** | **Outpatient visits model, N (%)** | | | |  | **Hospital admissions model, N (%)** | | | |  | **Unmet health needs model, N (%)** | | | |
| --- | --- | --- | --- | --- | --- | --- | --- | --- | --- | --- | --- | --- | --- | --- |
|  | **Total**  **(n =1140)** | **Private clinics**  **(n =523)** | **Public clinics**  **(n =617)** | ***P* value** |  | **Total**  **(n =1133)** | **Private clinics**  **(n =521)** | **Public clinics**  **(n =612)** | ***P* value** |  | **Total**  **(n =1140)** | **Private clinics**  **(n =521)** | **Public clinics**  **(n =619)** | ***P* value** |
| Gender |  |  |  |  |  |  |  |  |  |  |  |  |  |  |
| Male | 456(40.00) | 197(37.67) | 259(41.98) | 0.139 |  | 450(30.72) | 195(37.43) | 255(41.67) | 0.146 |  | 455(39.91) | 196(37.62) | 259(41.84) | 0.147 |
| Female | 684(60.00) | 326(62.33) | 358(58.02) |  |  | 683(60.28) | 326(62.57) | 357(58.33) |  |  | 685(60.09) | 325(62.38) | 360(58.16) |  |
| Age |  |  |  |  |  |  |  |  |  |  |  |  |  |  |
| 50–59 years old | 373(32.72) | 190(36.33) | 183(29.66) | 0..027 |  | 369(32.57) | 190(36.47) | 179(29.25) | 0.014 |  | 373(32.72) | 190(36.47) | 183(29.56) | 0.027 |
| 60–69 years old | 402(35.26) | 185(35.37) | 217(35.17) |  |  | 401(35.39) | 185(35.51) | 216(35.29) |  |  | 401(35.18) | 183(35.12) | 218(35.22) |  |
| 70–79 years old | 296(25.96) | 116(22.18) | 180(29.17) |  |  | 294(25.95) | 114(21.88) | 180(29.41) |  |  | 296(25.96) | 116(22.26) | 180(29.08) |  |
| ≥ 80 years old | 69(6.05) | 32(6.12) | 37(6.00) |  |  | 69(6.09) | 32(6.14) | 37(6.05) |  |  | 70(6.14) | 32(6.14) | 38(6.14) |  |
| Marital status |  |  |  |  |  |  |  |  |  |  |  |  |  |  |
| Single | 225(19.75) | 117(22.37) | 108(17.53) | 0.041 |  | 226(19.96) | 118(22.65) | 108(17.68) | 0.037 |  | 226(19.84) | 117(22.46) | 109(17.64) | 0.042 |
| Current partnership | 914(80.25) | 406(77.63) | 508(82.47) |  |  | 906(80.04) | 403(77.35) | 503(82.32) |  |  | 913(80.16) | 404(77.54) | 509(82.36) |  |
| Education |  |  |  |  |  |  |  |  |  |  |  |  |  |  |
| Illiterate | 387(33.95) | 208(39.77) | 179(29.01) | <0.001 |  | 385(33.98) | 207(39.73) | 178(29.08) | <0.001 |  | 386(33.86) | 206(39.54) | 180(29.08) | <0.001 |
| Primary school | 463(40.61) | 213(40.73) | 250(40.52) |  |  | 461(40.69) | 213(40.88) | 248(40.52) |  |  | 464(40.70) | 213(40.88) | 251(40.55) |  |
| Secondary school | 178(15.61) | 75(14.34) | 103(16.69) |  |  | 177(15.61) | 74(14.20) | 103(16.83) |  |  | 178(15.61) | 75(14.40) | 103(16.64) |  |
| High school or above | 112(9.82) | 27(5.16) | 85(13.78) |  |  | 110(9.71) | 27(5.18) | 83(13.56) |  |  | 112(9.82) | 27(5.18) | 85(13.73) |  |
| Residency |  |  |  |  |  |  |  |  |  |  |  |  |  |  |
| Urban | 422(37.02) | 167(31.93) | 255(41.33) | 0.001 |  | 419(36.98) | 165(31.67) | 254(41.50) | 0.001 |  | 424(37.19) | 167(32.05) | 257(41.52) | 0.001 |
| Rural | 718(62.98) | 356(68.07) | 362(58.67) |  |  | 714(63.02) | 356(68.33) | 358(58.50) |  |  | 716(62.81) | 354(67.95) | 362(58.48) |  |
| Insurance |  |  |  |  |  |  |  |  |  |  |  |  |  |  |
| No | 143(12.57) | 105(20.08) | 38(6.18) | <0.001 |  | 143(12.64) | 105(20.15) | 38(6.23) | <0.001 |  | 143(12.57) | 105(20.15) | 38(6.16) | <0.001 |
| Yes | 995(87.43) | 418(79.92) | 577(93.82) |  |  | 988(87.36) | 416(79.85) | 572(93.77) |  |  | 995(87.43) | 416(79.85) | 579(93.84) |  |
| Income quintile |  |  |  |  |  |  |  |  |  |  |  |  |  |  |
| Poorest | 300(26.36) | 178(34.10) | 122(19.81) | <0.001 |  | 300(26.53) | 178(34.23) | 122(19.97) | <0.001 |  | 300(26.36) | 178(34.23) | 122(19.74) | <0.001 |
| Q2 | 274(24.08) | 156(29.89) | 118(19.16) |  |  | 272(24.05) | 154(29.62) | 118(19.31) |  |  | 274(24.08) | 155(29.81) | 119(19.26) |  |
| Q3 | 216(19.98) | 85(16.28) | 131(21.27) |  |  | 215(19.01) | 85(16.35) | 130(21.28) |  |  | 216(18.95) | 84(16.15) | 132(21.36) |  |
| Q4 | 210(18.45) | 76(14.56) | 134(21.75) |  |  | 207(18.30) | 76(14.62) | 131(21.44) |  |  | 210(18.45) | 76(14.62) | 134(21.68) |  |
| Richest | 138(12.13) | 27(5.17) | 111(18.02) |  |  | 137(12.11) | 27(5.19) | 110(18.00) |  |  | 138(12.13) | 27(5.19) | 111(17.96) |  |
| BMI |  |  |  |  |  |  |  |  |  |  |  |  |  |  |
| Underweight | 30(2.63) | 15 (2.87) | 15(2.43) | 0.078 |  | 30(2.65) | 15(2.88) | 15(2.45) | 0.119 |  | 30(2.63) | 15(2.88) | 15(2.42) | 0.097 |
| Normal weight | 565(49.56) | 277 (52.96) | 288(46.68) |  |  | 565(49.87) | 276(52.98) | 289(47.22) |  |  | 565(49.56) | 275(52.78) | 290(46.85) |  |
| Overweight | 402(35.26) | 177(33.84) | 225(36.47) |  |  | 379(35.04) | 176(33.78) | 221(36.11) |  |  | 402(35.26) | 177(34.97) | 225(36.35) |  |
| Obesity | 143(12.54) | 54(10.33) | 89(14.42) |  |  | 141(12.44) | 54(10.36) | 87(14.22) |  |  | 143(12.54) | 54(10.36) | 89(14.38) |  |
| ADLs |  |  |  |  |  |  |  |  |  |  |  |  |  |  |
| No | 275(24.12) | 99(18.93) | 176(28.53) | <0.001 |  | 272(24.01) | 98(18.81) | 174(28.43) | <0.001 |  | 276(24.21) | 99(19.00) | 177(28.59) | <0.001 |
| Yes | 865(75.88) | 424(81.07) | 441(71.47) |  |  | 861(75.99) | 423(81.19) | 438(71.57) |  |  | 864(75.79) | 422(81.00) | 442(71.41) |  |
| IADLs |  |  |  |  |  |  |  |  |  |  |  |  |  |  |
| No | 988(86.67) | 453(86.62) | 535(86.71) | 0.963 |  | 983(86.76) | 452(86.76) | 531(86.76) | 0.997 |  | 988(86.67) | 451(86.56) | 537(86.57) | 0.926 |
| Yes | 152(13.33) | 70(13.38) | 82(13.29) |  |  | 150(13.24) | 69(13.12) | 81(13.24) |  |  | 152(13.33) | 70(13.44) | 82(13.25) |  |
| Depression |  |  |  |  |  |  |  |  |  |  |  |  |  |  |
| No | 1107(97.11) | 505(96.56) | 602(97.57) | 0.311 |  | 1100(97.09) | 503(96.55) | 597(97.55) | 0.317 |  | 1107(97.11) | 503(96.55) | 604(97.58) | 0.301 |
| Yes | 33(2.89) | 18(3.44) | 15(2.43) |  |  | 33(2.91) | 18(3.45) | 15(2.45) |  |  | 33(2.89) | 18(3.45) | 15(2.42) |  |
| Multimorbidity |  |  |  |  |  |  |  |  |  |  |  |  |  |  |
| No | 553(48.51) | 258(49.33) | 295(47.81) | 0.609 |  | 550(48.54) | 259(49.71) | 291(47.55) | 0.468 |  | 553(48.51) | 258(49.52) | 295(47.66) | 0.531 |
| Yes | 587(51.49) | 265(50.67) | 322(52.19) |  |  | 583(51.46) | 262(50.29) | 321(52.45) |  |  | 587(51.49) | 263(50.48) | 324(52.34) |  |

^a^ Distribution reported excluded those with missing data. USC, usual source of care; BMI, body mass index; ADLs, activities of daily living; IADLs, instrumental activities of daily living limitation; N, number.

Table S3 The mean value of outpatient visits among different types of USCs ^a^ (China, 2010).

| **Characteristics** | **Public hospitals** | **Primary care facilities** | **P value** |  | **Private clinics** | **Public clinics** | **P value** |
| --- | --- | --- | --- | --- | --- | --- | --- |
| Gender |  |  |  |  |  |  |  |
| Male | 5.29 | 3.63 | 0.005 |  | 2.18 | 4.73 | 0.400 |
| Female | 7.02 | 3.54 |  |  | 2.69 | 4.31 |  |
| Age |  |  |  |  |  |  |  |
| 50–59 years old | 4.86 | 3.61 | 0.019 |  | 2.95 | 4.30 | 0.804 |
| 60–69 years old | 5.92 | 3.47 |  |  | 2.25 | 4.51 |  |
| 70–79 years old | 6.81 | 3.66 |  |  | 2.38 | 4.48 |  |
| ≥ 80 years old | 9.92 | 3.65 |  |  | 1.66 | 5.38 |  |
| Marital status |  |  |  |  |  |  |  |
| Single | 7.59 | 3.56 | 0.093 |  | 2.44 | 4.79 | 0.738 |
| Current partnership | 5.95 | 3.58 |  |  | 2.51 | 4.44 |  |
| Education |  |  |  |  |  |  |  |
| Illiterate | 6.24 | 3.49 | 0.0001 |  | 2.91 | 4.17 | 0.183 |
| Primary school | 4.84 | 3.30 |  |  | 2.16 | 4.28 |  |
| Secondary school | 6.54 | 3.46 |  |  | 2.44 | 4.19 |  |
| High school or above | 7.61 | 5.18 |  |  | 2.11 | 6.15 |  |
| Residency |  |  |  |  |  |  |  |
| Urban | 7.06 | 3.67 | 0.069 |  | 2.06 | 4.73 | 0.194 |
| Rural | 3.78 | 3.52 |  |  | 2.70 | 4.32 |  |
| Insurance |  |  |  |  |  |  |  |
| No | 2.65 | 2.72 | <0.001 |  | 2.16 | 4.26 | <0.001 |
| Yes | 6.85 | 3.67 |  |  | 2.58 | 4.46 |  |
| Income quintile |  |  |  |  |  |  |  |
| Poorest | 3.00 | 2.66 | 0.0001 |  | 2.51 | 2.88 | 0.0001 |
| Q2 | 3.66 | 2.77 |  |  | 2.58 | 3.02 |  |
| Q3 | 4.83 | 3.86 |  |  | 1.66 | 5.28 |  |
| Q4 | 6.90 | 5.35 |  |  | 3.25 | 6.54 |  |
| Richest | 9.49 | 3.83 |  |  | 2.59 | 4.13 |  |
| BMI |  |  |  |  |  |  |  |
| Underweight | 4.36 | 4.60 | 0.006 |  | 2.60 | 6.60 | 0.176 |
| Normal weight | 6.05 | 3.39 |  |  | 2.28 | 4.45 |  |
| Overweight | 6.53 | 3.25 |  |  | 2.73 | 3.66 |  |
| Obesity | 6.60 | 5.02 |  |  | 2.83 | 6.35 |  |
| ADLs |  |  |  |  |  |  |  |
| No | 7.52 | 3.40 | 0.022 |  | 2.44 | 3.93 | 0.892 |
| Yes | 5.72 | 3.63 |  |  | 2.51 | 4.71 |  |
| IADLs |  |  |  |  |  |  |  |
| No | 6.24 | 3.40 | 0.057 |  | 2.41 | 4.25 | 0.001 |
| Yes | 6.42 | 4.68 |  |  | 3.09 | 6.05 |  |
| Depression |  |  |  |  |  |  |  |
| No | 6.27 | 3.57 | 0.750 |  | 2.46 | 4.50 | 0.975 |
| Yes | 5.12 | 3.70 |  |  | 3.56 | 3.87 |  |
| Multimorbidity |  |  |  |  |  |  |  |
| No | 4.70 | 2.80 | <0.001 |  | 1.86 | 3.63 | 0.007 |
| Yes | 7.25 | 4.30 |  |  | 3.12 | 5.27 |  |

USC, usual source of care; BMI, body mass index; ADLs, activities of daily living; IADLs, instrumental activities of daily living limitation. ^a^ Rank sum test was used.

Table S4 The mean value of hospital admissions among different types of USCs ^a^ (China, 2010).

| **Characteristics** | **Public hospitals** | **Primary care facilities** | **P value** |  | **Private clinics** | **Public clinics** | **P value** |
| --- | --- | --- | --- | --- | --- | --- | --- |
| Gender |  |  |  |  |  |  |  |
| Male | 0.32 | 0.20 | 0.064 |  | 0.22 | 0.19 | 0.266 |
| Female | 0.31 | 0.17 |  |  | 0.18 | 0.17 |  |
| Age |  |  |  |  |  |  |  |
| 50–59 years old | 0.24 | 0.15 | 0.003 |  | 0.18 | 0.12 | 0.822 |
| 60–69 years old | 0.29 | 0.20 |  |  | 0.21 | 0.19 |  |
| 70–79 years old | 0.40 | 0.20 |  |  | 0.21 | 0.20 |  |
| ≥ 80 years old | 0.29 | 0.19 |  |  | 0.13 | 0.24 |  |
| Marital status |  |  |  |  |  |  |  |
| Single | 0.34 | 0.22 | 0.248 |  | 0.21 | 0.22 | 0.281 |
| Current partnership | 0.31 | 0.18 |  |  | 0.19 | 0.17 |  |
| Education |  |  |  |  |  |  |  |
| Illiterate | 0.18 | 0.20 | 0.790 |  | 0.20 | 0.20 | 0.052 |
| Primary school | 0.33 | 0.16 |  |  | 0.18 | 0.14 |  |
| Secondary school | 0.29 | 0.24 |  |  | 0.28 | 0.20 |  |
| High school or above | 0.29 | 0.15 |  |  | 0.07 | 0.18 |  |
| Residency |  |  |  |  |  |  |  |
| Urban | 0.30 | 0.17 | 0.824 |  | 0.14 | 0.19 | 0.420 |
| Rural | 0.37 | 0.19 |  |  | 0.22 | 0.17 |  |
| Insurance |  |  |  |  |  |  |  |
| No | 0.26 | 0.12 | 0.033 |  | 0.14 | 0.05 | 0.069 |
| Yes | 0.32 | 0.19 |  |  | 0.21 | 0.18 |  |
| Income quintile |  |  |  |  |  |  |  |
| Poorest | 0.35 | 0.22 | 0.885 |  | 0.22 | 0.22 | 0.774 |
| Q2 | 0.35 | 0.16 |  |  | 0.16 | 0.16 |  |
| Q3 | 0.32 | 0.21 |  |  | 0.19 | 0.22 |  |
| Q4 | 0.32 | 0.17 |  |  | 0.25 | 0.12 |  |
| Richest | 0.28 | 0.14 |  |  | 0.15 | 0.14 |  |
| BMI |  |  |  |  |  |  |  |
| Underweight | 0.29 | 0.23 | 0.240 |  | 0.20 | 0.27 | 0.821 |
| Normal weight | 0.34 | 0.18 |  |  | 0.20 | 0.16 |  |
| Overweight | 0.27 | 0.18 |  |  | 0.19 | 0.17 |  |
| Obesity | 0.36 | 0.21 |  |  | 0.20 | 0.22 |  |
| ADLs |  |  |  |  |  |  |  |
| No | 0.20 | 0.11 | <0.001 |  | 0.16 | 0.08 | 0.002 |
| Yes | 0.36 | 0.21 |  |  | 0.20 | 0.21 |  |
| IADLs |  |  |  |  |  |  |  |
| No | 0.29 | 0.16 | <0.001 |  | 0.17 | 0.14 | 0.0001 |
| Yes | 0.57 | 0.36 |  |  | 0.33 | 0.38 |  |
| Depression |  |  |  |  |  |  |  |
| No | 0.30 | 0.18 | 0.003 |  | 0.19 | 0.17 | 0.131 |
| Yes | 0.78 | 0.27 |  |  | 0.22 | 0.33 |  |
| Multimorbidity |  |  |  |  |  |  |  |
| No | 0.20 | 0.14 | <0.001 |  | 0.15 | 0.12 |  |
| Yes | 0.38 | 0.23 |  |  | 0.24 | 0.22 | 0.0005 |

USC, usual source of care; BMI, body mass index; ADLs, activities of daily living; IADLs, instrumental activities of daily living limitation. ^a^ Rank sum test was used.

Table S5 The probability of unmet health needs by different types of USCs [%] ^a^ (China, 2010).

| **Characteristics** | **Public hospitals** | **Primary care facilities** | **P value** |  | **Private clinics** | **Public clinics** | **P value** |
| --- | --- | --- | --- | --- | --- | --- | --- |
| Gender |  |  |  |  |  |  |  |
| Male | 4.41 | 11.43 | 0.747 |  | 15.82 | 8.11 | 0.626 |
| Female | 4.03 | 10.51 |  |  | 16.31 | 5.28 |  |
| Age |  |  |  |  |  |  |  |
| 50–59 years old | 5.79 | 14.21 | 0.002 |  | 22.63 | 5.46 | 0.075 |
| 60–69 years old | 3.77 | 9.48 |  |  | 13.66 | 5.96 |  |
| 70–79 years old | 3.81 | 8.45 |  |  | 10.34 | 7.22 |  |
| ≥ 80 years old | 2.13 | 11.43 |  |  | 12.50 | 10.53 |  |
| Marital status |  |  |  |  |  |  |  |
| Single | 3.97 | 7.52 | 0.170 |  | 10.26 | 4.59 | 0.076 |
| Current partnership | 4.25 | 11.61 |  |  | 17.82 | 6.68 |  |
| Education |  |  |  |  |  |  |  |
| Illiterate | 5.72 | 11.14 | 0.001 |  | 14.08 | 7.78 | 0.390 |
| Primary school | 3.89 | 12.28 |  |  | 18.31 | 7.17 |  |
| Secondary school | 5.17 | 8.43 |  |  | 14.67 | 3.88 |  |
| High school or above | 2.66 | 8.04 |  |  | 18.52 | 4.71 |  |
| Residency |  |  |  |  |  |  |  |
| Urban | 4.90 | 10.85 | 0.264 |  | 17.37 | 6.61 | 0.981 |
| Rural | 2.04 | 10.89 |  |  | 15.54 | 6.35 |  |
| Insurance |  |  |  |  |  |  |  |
| No | 12.68 | 19.58 | 0.000 |  | 20.95 | 15.79 | 0.000 |
| Yes | 2.81 | 9.65 |  |  | 14.90 | 5.87 |  |
| Income quintile |  |  |  |  |  |  |  |
| Poorest | 12.40 | 14.67 | 0.000 |  | 17.98 | 9.84 | 0.002 |
| Q2 | 3.79 | 14.23 |  |  | 18.06 | 9.24 |  |
| Q3 | 3.07 | 8.33 |  |  | 14.29 | 4.55 |  |
| Q4 | 2.86 | 6.19 |  |  | 10.53 | 3.73 |  |
| Richest | 2.95 | 6.52 |  |  | 11.11 | 5.41 |  |
| BMI |  |  |  |  |  |  |  |
| Underweight | 6.38 | 13.33 | 0.676 |  | 20.00 | 6.67 | 0.973 |
| Normal weight | 4.19 | 10.97 |  |  | 14.91 | 7.24 |  |
| Overweight | 4.49 | 10.70 |  |  | 17.51 | 5.33 |  |
| Obesity | 3.11 | 10.49 |  |  | 16.67 | 6.74 |  |
| ADLs |  |  |  |  |  |  | 0.181 |
| No | 3.07 | 8.70 | 0.011 |  | 15.15 | 5.08 |  |
| Yes | 4.68 | 11.57 |  |  | 16.35 | 7.01 |  |
| IADLs |  |  |  |  |  |  |  |
| No | 4.22 | 11.44 | 0.381 |  | 17.07 | 6.70 | 0.122 |
| Yes | 4.00 | 7.24 |  |  | 10.00 | 4.88 |  |
| Depression |  |  |  |  |  |  |  |
| No | 4.05 | 10.84 | 0.045 |  | 16.30 | 6.29 | 0.816 |
| Yes | 12.20 | 12.12 |  |  | 11.11 | 13.33 |  |
| Multimorbidity |  |  |  |  |  |  |  |
| No | 3.03 | 11.57 | 0.831 |  | 18.99 | 5.08 | 0.464 |
| Yes | 4.95 | 10.22 |  |  | 13.31 | 7.72 |  |

USC, usual source of care; BMI, body mass index; ADLs, activities of daily living; IADLs, instrumental activities of daily living limitation. ^a^ Chi-square test was used.

Table S6 Standardized differences between different types of USCs (public hospitals and primary care facilities) before and after weighting [%] (China, 2010).

| **Characteristics** | **Outpatient visits model** | |  | **Hospital admissions model** | |  | **Unmet health needs model** | |
| --- | --- | --- | --- | --- | --- | --- | --- | --- |
|  | **Before** | **After** |  | **Before** | **After** |  | **Before** | **After** |
| Gender | 9.30 | -0.40 |  | 9.98 | -0.68 |  | 9.57 | -0.32 |
| Age | -17.61 | -0.72 |  | -17.61 | -0.92 |  | -17.95 | -0.83 |
| Marital status | -3.62 | -0.37 |  | -4.24 | -0.42 |  | -3.88 | -0.41 |
| Education | -55.00 | -0.83 |  | -55.62 | -0.81 |  | -55.02 | -1.01 |
| Residency | 84.66 | -0.28 |  | 85.46 | -0.31 |  | 84.65 | -0.24 |
| Insurance | 2.02 | 1.90 |  | 0.86 | 1.92 |  | 1.69 | 1.89 |
| Income quintile | -58.24 | 1.34 |  | -59.56 | 1.34 |  | -58.61 | 1.20 |
| BMI | -1.40 | -1.01 |  | -3.05 | -0.70 |  | -1.62 | -1.07 |
| ADLs | 12.61 | -0.31 |  | 13.33 | -0.44 |  | 12.46 | -0.32 |
| IADLs | 13.49 | -1.10 |  | 13.44 | -1.29 |  | 13.44 | -1.24 |
| Depression | 6.50 | -0.38 |  | 6.49 | -0.32 |  | 6.62 | -0.35 |
| Multimorbidity | -19.02 | 1.50 |  | -19.18 | 1.21 |  | -19.21 | 1.46 |

USC, usual source of care; BMI, body mass index; ADLs, activities of daily living; IADLs, instrumental activities of daily living limitation.

Table S7 Standardized differences between different types of USCs (private clinics and public clinics) before and after weighting [%] (China, 2010).

| **Characteristics** | **Outpatient visits model** | |  | **Hospital admissions model** | |  | **Unmet health needs model** | |
| --- | --- | --- | --- | --- | --- | --- | --- | --- |
|  | **Before** | **After** |  | **Before** | **After** |  | **Before** | **After** |
| Gender | -8.74 | 1.93 |  | -8.60 | 2.19 |  | -8.56 | 2.04 |
| Age | 14.32 | -4.65 |  | 15.59 | -4.76 |  | 14.62 | -4.62 |
| Marital status | 12.50 | 2.39 |  | 12.79 | 2.40 |  | 12.43 | 2.35 |
| Education | 32.87 | 2.53 |  | 32.60 | 2.18 |  | 32.30 | 2.41 |
| Residency | -19.20 | -0.30 |  | -20.12 | 0.04 |  | -19.32 | -0.24 |
| Insurance | 42.70 | -0.56 |  | 42.70 | -0.83 |  | 42.97 | -0.55 |
| Income quintile | 55.54 | 3.35 |  | 54.75 | 3.37 |  | 55.49 | 3.34 |
| BMI | 14.96 | 1.26 |  | 13.87 | 1.15 |  | 14.41 | 1.21 |
| ADLs | -22.90 | -1.43 |  | -23 | -1.19 |  | -22.87 | -1.45 |
| IADLs | -0.16 | -2.30 |  | 0.09 | -2.15 |  | -0.44 | -2.28 |
| Depression | -5.94 | 2.47 |  | -5.89 | 2.59 |  | -6.07 | 2.47 |
| Multimorbidity | 2.70 | -0.75 |  | 3.99 | -0.74 |  | 3.39 | -0.80 |

USC, usual source of care; BMI, body mass index; ADLs, activities of daily living; IADLs, instrumental activities of daily living limitation.

Table S8 Subgroup analyses for the health care utilization between different types of USCs and residency after weighting (China, 2010).

| **Characteristics** | | **Outpatient visits model** | |  | **Hospital admissions model** | |  | **Unmet health needs model** | |
| --- | --- | --- | --- | --- | --- | --- | --- | --- | --- |
|  |  | ***IRR (95% CI)*** | ***P* Value** |  | ***IRR (95% CI)*** | ***P* Value** |  | ***OR (95% CI)*** | ***P* Value** |
| Rural area | USC (ref. = public hospitals) |  |  |  |  |  |  |  |  |
|  | Primary care facilities | 0.973(0.734,1.289) | 0.841 |  | 0.516(0.406,0.655) | <0.001 |  | 3.522（1.067,11.629） | 0.040 |
|  | USC (ref. = private clinics) |  |  |  |  |  |  |  |  |
|  | Public clinics | 1.965(1.494,2.586) | <0.001 |  | 0.613(0.389,0.967) | 0.036 |  | 0.766（0.397,1.476） | 0.410 |
| Urban area | USC (ref. = public hospitals) |  |  |  |  |  |  |  |  |
|  | Primary care facilities | 0.923(0.576,1.414) | 0.642 |  | 0.505(0.361,0.706) | <0.001 |  | 1.108（0.717,1.711） | 0.632 |
|  | USC (ref. = private clinics) |  |  |  |  |  |  |  |  |
|  | Public clinics | 2.423(1.293,4.542) | 0.008 |  | 2.629(1.328,5.204) | 0.008 |  | 0.628（0.242,1.629） | 0.322 |

USC, usual source of care; *IRR*, adjusted relative rate; *OR*, odds ratio.
